# Biosorption of Copper (II) from Aqueous Solution Using Non-Living *Mesorhizobium amorphae* Strain CCNWGS0123

**DOI:** 10.1264/jsme2.ME11331

**Published:** 2012-02-22

**Authors:** Osama Abdalla Mohamad, Xiuli Hao, Pin Xie, Shaimaa Hatab, Yanbing Lin, Gehong Wei

**Affiliations:** 1 College of Life Sciences, State Key Laboratory of Crop Stress Biology in Arid Areas, Northwest A&F University, Yangling, Shaanxi 712100, China; 2 College of Resources and Environment, Northwest A&F University, Yangling, Shaanxi 712100, China; 3 College of Food Science and Engineering, Northwest A&F University, Yangling, Shaanxi 712100, China

**Keywords:** biosorption, copper, dead, *Mesorhizobium*, environment

## Abstract

The mining industry generates huge amounts of wastewater, containing toxic heavy metals. Treatment to remove heavy metals is necessary and recent work has been focused on finding more environmentally friendly materials for removing heavy metals from wastewater. Biosorption can be an effective process for heavy metal removal from aqueous solutions. Our objectives were to investigate the removal of copper (II) from aqueous solutions using dead cells of *Mesorhizobium amorphae* CCNWGS0123 under differing levels of pH, agitation speed, temperature, initial copper concentration, biosorbent dose and contact time using flame atomic absorption spectroscopy for metal estimation. The maximum copper removal rate was achieved at pH 5.0, agitation speed 150×*g*, temperature 28°C and initial Cu (II) concentration of 100 mg L^−1^. Maximum biosorption capacity was at 0.5 g L^−1^ and equilibrium was attained within 30 min. Langmuir and Freundlich isotherms showed correlation coefficients of 0.958 and 0.934, respectively. Fourier transform-infrared spectroscopy (FT-IR) analysis indicated that many functional groups, such as O-H, N-H, C-H, C=O, -NH, -CN, C-N, C-O, amide -I, -II, -III and unsaturated alkenes, alkyls and aromatic groups on the cell surface were involved in the interaction between CCNWGS0123 and Cu. Scanning electron microscope and energy dispersive X-ray scanning results showed deformation, aggregation, and cell-surface damage due to the precipitation of copper on the cell surface. Dead cells of CCNWGS0123 showed potential as an efficient biosorbent for the removal of Cu^2+^ from aqueous solutions.

Toxic metal contamination from mining operations is an important environmental concern. Soil contamination by toxic metals comes from anthropogenic sources, such as smelters, mining, power stations, and the application of pesticides containing metal, fertilizer, and sewage sludge (26). Mining operation by-products (tailings) can contain high concentrations of Cu, Zn, Fe, Mn, Ni, Pb and Cd, ranging from 1 to 150 g kg^−1^(25).

Copper (Cu) is an important trace component of a variety of oxidases, such as ascorbic acid oxidase, polyphenol oxidase, and superoxide dismutase along with electronic transfer proteins involved in many redox reactions (3). Indeed, copper is a useful metal in a broad range of industrial applications, such as an active ingredient in catalysts, pigments, pesticides, fertilizers, and stabilizers for PVC (41); however, at certain levels Cu can be toxic, polluting soil and water, and has the potential to bioaccumulate in living tissues. Toxic levels of Cu have led the United States Environmental Protection Agency (USEPA) to specify a threshold copper aqueous concentration limit, defined as a criteria maximum contaminant level (CMC) of 13 μg L^−1^(36). In addition, the World Health Organization (WHO) has recommended a maximum acceptable concentration of Cu in drinking water of 1.5 mg L^−1^(27).

Certain types of microbial biomass can retain relatively high quantities of metals. This can be dependent on the affinity between the metallic element or its ionic forms and the binding sites on the molecular structure of the cellular wall (28). Binding sites are present in cell walls and are composed of lipopolysaccharides, peptidoglycans and phospholipids. They are also present in extracellular polymer substances (EPS), which are composed of neutral sugar compounds such as galactose and glucose, with minor amounts of mannose, xylose, arabinose, rhamnose, fructose and two O-methyl sugars (42).

During the last decade, much research has focused on economical and efficient treatment processes that facilitate the removal of toxic elements from wastewater, and a promising strategy involves immobilizing systems of micro-organisms, such as bacteria, fungi or algae, in polymeric matrices suitable for metal ion uptake applications (24, 38). Biosorption, based on live or dead biosorbents, has been regarded as a cost-effective biotechnology for the treatment of high volumes of complex wastewater containing toxic metals at low concentrations (8). It has been reported that dead cells have higher efficiency for metal biosorption than living cells (21, 24, 30). This is due to the fact that living cells produce redox reactions between the cells and aqueous medium, causing an increase in final pH. In addition, living systems (active uptake) often require the addition of nutrients, which increase the biological oxygen demand or chemical oxygen demand in the effluent (16). Moreover, using dead cells has many advantages: the metal removal system is not subject to metal toxicity limitations, and there is no requirement for growth media and nutrients. Also, sorbed metal ions can be easily desorbed and the biomass can be reused (13). We chose non-living *Mesorhizobium amorphae* strain CCNWGS0123 for the biosorption of Cu (II) as an alternative treatment of wastewater because it is easy to manipulate, and dead cells have better performance and are cheaper than live cells.

Seventy-six bacterial strains were isolated from root nodules collected from plants growing in tailings from the Taibai gold mining region, Gansu province, China. CCNWGS0123 is a Gram-negative soil bacterium that has profound scientific and agronomic significance due to its ability to establish nitrogen-fixing symbioses with leguminous plants, which is of major importance to the maintenance of soil fertility (32). The objectives of the present work were to investigate the biosorption potential of dead cells and the influence on biosorption of different Cu^2+^ biosorption parameters, such as the initial concentration of Cu^2+^, pH, agitation speed, contact time and biosorption dose. Furthermore, in order to study the biosorption mechanism before and after the biosorption process, FT-IR analyses were used to study potential binding sites and possible functional groups on cell walls. Scanning electron microscopy (SEM) coupled with energy dispersive X-ray (EDX) scanning was used to study the surface structure and cell morphology. To our knowledge, this is the first time that dead cells of CCNWGS0123 have been used as absorbents of copper ions. They can be classified according to the 16S rRNA phylogenetic tree in the branch of *Mesorhizobium amorphae* (Fig. 1).

## Materials and Methods

### Isolation of *Rhizobium* strains from contaminated soils

Root nodules of *Robinia pseudoacacia* were collected from rhizosphere soil in tailings from the Taibai gold mine region, Gansu Province, northwestern China. Yeast-mannitol agar (YMA) medium (3 g yeast extract, 10 g mannitol, 0.25 g K_2_HPO_4_·5H_2_O, 0.25 g KH_2_PO_4_, 0.2 g MgSO_4_·7H_2_O, 0.1 g NaCl, and 20 g agar per liter) was used for the growth of bacteria (39). Single colonies were selected and checked for purity by repeated streaking and microscopic examination. All isolates were incubated at 28°C and maintained on YMA slants either at 4°C or in 20% (v/v) glycerol solution at −70°C.

### Screening test for heavy metal-resistant strains

To isolate Cu^2+^-resistant strains, samples were screened on YMA plates supplemented with copper at concentrations ranging from 0.1 to 1.0 mM CuSO_4_·5H_2_O. Using multi-point inoculators, a 200 μL aliquot of cell suspension from each sample was added to the YMA plates and the cultures were incubated at 28°C for 3 to 5 days. In order to confirm the results of the screening test, strain CCNWGS0123 was re-inoculated into 5 ml tryptone yeast (TY) liquid medium (5 g tryptone, 3 g yeast extract, and 0.7 g CaCl_2_·2H_2_O per liter), supplemented with copper at concentrations ranging from 0.1 to 4.0 mM: CuSO_4_·5H_2_O. The cultures were incubated at 28°C and agitated at 150×*g* for 3 to 5 days. The growth values of the strains were determined by absorbance at 600 nm (OD_600_). All treatments in this study were replicated three times and mean values and standard errors were calculated using Excel (Microsoft Office, 2007). Also, blank experiments were conducted to ensure that no absorption occurred on the walls of the apparatus.

### Preparation of the bacterial biosorbents and copper stock solution

Strain CCNWGS0123 was pre-cultured in TY medium at 28°C for 72 h with shaking at 150×*g*. At the end of the exponential phase, cells were harvested by centrifugation at 12,000×*g* for 15 min. Collected cells were rinsed three times with sterilized deionized distilled water (ddH_2_O) and then subjected to autoclaving at 121°C for 20 min (21). CCNWGS0123 was re-suspended in the designated toxic-metal solutions for biosorption experiments.

Copper standard stock solution of 1.0 mg L^−1^ was prepared from a ready-made standard (SIMT, Shanghai, China). Working Cu solutions were prepared by diluting the stock solution to the desired appropriate ratio right before each biosorption experiment.

### Effect of initial copper concentration and biosorption dose

Effects of different initial copper concentration and the biosorbent dose on copper biosorption capacity and the removal rate by dead cells of CCNWGS0123 were examined in order to determine the optimum biosorption conditions. All samples were agitated at 150×*g* and 28°C for 24 h in an initial Cu^2+^ concentration ranging from 50 to 500 mg L^−1^ Cu^2+^ with biosorption doses ranging from 0.5 to 2.5 g L^−1^ dry weight. The residual Cu ions in the supernatant were measured by an atomic absorption spectrophotometer-AAS (Z-5000; Hitachi, Tokyo, Japan) after centrifugation at 10,000×*g* for 10 min. The values of the biosorption capacity and removal rate of Cu^2+^ were evaluated as follows (6).

$$\begin{array}{l} q_{e}=(C_{0}-C_{e})/B\\ \text{Removal rate }(\%)=\frac{{\text{C}}_{0}-{\text{C}}_{\text{e}}}{{\text{C}}_{0}}\times 100 \end{array}$$

Where q_e_ is the equilibrium Cu^2+^ concentration on the biosorbent (mg g^−1^ dry cell); C_0_ is the initial metal ion concentration mg L^−1^, C_e_ is the residual metal concentration in solution at equilibrium with the biosorbent (mg L^−1^), and B is the biomass concentration (g L^−1^ dry cell).

### Effect of pH, agitation speed and temperature on biosorption process

Variations in pH, agitation speed and temperature on Cu^2+^ biosorption were studied with an initial concentration of 100 mg L^−1^ Cu^2+^ and biosorption dose of 0.5 g L^−1^. The pH was adjusted from 1 to 6, measured with a digital HANNA pH-211 meter (Shanghai, China) by the addition of predetermined amounts of sterilized 0.1 M HCl and NaOH. The agitation speeds were varied from 60 to 210×*g*. Temperature was adjusted from 22 to 37°C. After 24 h, the supernatant was harvested by centrifuging (10,000×*g* for 10 min) and the metal ion concentrations were detected immediately with AAS.

### Biosorption isotherm

To determine the biosorption equilibrium of Cu (II) at a dosage of 0.5 g L^−1^, the biosorbent was suspended in Cu^2+^ solution with an initial concentration ranging from 50 to 500 mg L^−1^. The residual metal concentration in the suspension was measured after 24 h incubation at 28°C and 150×*g* agitation speed. Langmuir (20) and Freundlich (12) isotherms were used to determine the characteristic parameters of biosorption simulated in the experiments.

### Time course of biosorption

The bacterial biosorbent was suspended in 200 mL dd H_2_O containing 100 mg L^−1^ Cu^2+^ solution in a 500 mL Erlenmeyer flask with a bacterial concentration of 0.5 g L^−1^. pH was adjusted to 5.0 in order to avoid the precipitation of metals to metal hydroxides (4). The suspension was agitated at 150×*g* and 28°C. Samples were taken from the solution every 5 min for the first hour and then every 60 min up to 600 min. Samples were centrifuged at 10,000×*g* for 10 min. The Cu ions in the supernatant were measured by AAS to determine the residual metal concentration (C_e_ mg L^−1^) and the biosorption capacity q_e_ (mg g^−1^ dry cell) was calculated (6).

### Study with Fourier Transform Infrared (FT-IR) spectroscopy

Infrared spectra of dead biosorbent loaded with and without copper ions were determined at bacterial concentrations of 0.5 g L^−1^ and 100 mg L^−1^ Cu^2+^. Infrared analysis was performed with a 330 FT-IR (Fourier transform–infrared spectrometer; Nicolet Avatar; Nicolet, Pittsfield, MA, USA) at a range of 4,000 to 400 cm^−1^. One-milligram powder of dry weight cells was mixed and ground with 100 mg KBr in an agate mortar in order to investigate the functional groups and possible Cu binding site related to Cu biosorption.

### Scanning Electron Microscopy (SEM) analysis and Energy Dispersive X-ray (EDX) scanning

The surface structure and cell morphology of biosorbent after being loaded with 100 mg L^−1^ Cu^2+^ were observed at different magnifications by SEM (JSM-6360 LV; JEOL, Peabody, MA, USA) coupled with EDX. The cells were fixed with 3% glutaraldehyde for 24 h followed by dehydration with a graded series of ethanol concentrations (30, 50, 70, 80, 90 and 100%) for 30 min each. Cells were then dried in a critical-point dryer using CO_2_ (K850; EMITECH, East Grinstead, UK). The metal-loaded samples were mounted on a stainless steel slab with double-sided adhesive tape with a thin layer of gold in a high vacuum. Specimens were then examined with SEM/EDX.

### Identification of the strain by a molecular method

Strain CCNWGS0123 was incubated in TY broth medium for 3 days at 28°C with shaking at 150×*g*. Total DNA was extracted following the protocol of Sambrook and Russell (29). Total genomic DNA was isolated as described previously and used as a PCR template. PCR amplification was performed with Go Taq kit according to the manufacturer’s recommendations (Promega, Munich, Germany). Primers and PCR conditions are described in Table 1(17, 19, 33, 40).

### DNA sequencing of PCR products and sequence analysis

Restriction fragments were separated by electrophoresis in 1% agarose gel stained with ethidium bromide and visualized using a Bio-Rad UV-transilluminator. PCR bands were excised from the gel and DNA was extracted using HQ-20 PCR DNA and a Gel Band purification Kit (Anhui U-gene Biotechnology). After purification, the PCR product was sequenced directly using an ABI PRISM 377 DNA sequence analyzer (Perkin-Elmer Applied Biosystems, CA, USA). The nucleotide sequence and deduced protein sequences were compared with sequences in Gene Bank using BLAST (http://blast.ncbi.nlm.nih.gov/Blast.cgi) and the strain was identified by the sequence.

### Nucleotide sequence accession numbers

Sequences of 16S rRNA, *nodA*, *nodC and nifH* gene fragments in this study are available in the National Center for Biotechnology Information (NCBI Gene Bank database) under accession numbers JF907683, JF907684, JF907685 and JF907686.

## Results and Discussion

### Screening test

Results confirmed that CCNWGS0123 could grow in YMA medium supplemented with 0.6 mM Cu^2+^, TY medium 2.2 mM. *M. amorphae* showed high resistance to Cu (II); therefore, it was used in subsequent studies.

### Initial copper concentration

**Fig. 2:**
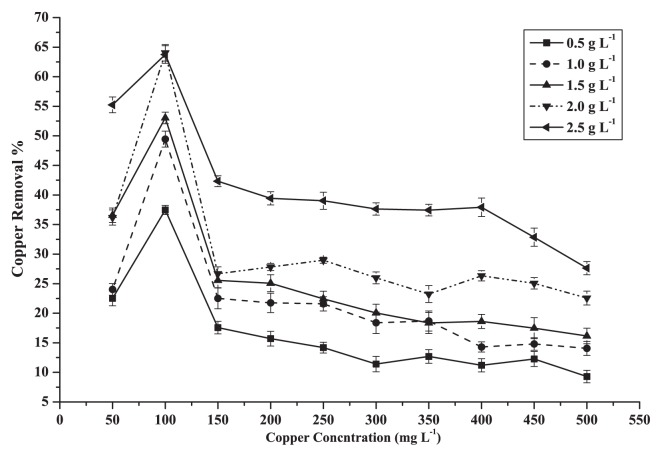
Removal efficiency of Cu^2+^ by dead cells of CCNWGS0123 over initial concentration ranging from 50 to 500 mg L^−1^ with the biosorbent dose ranging from 0.5 to 2.5 g L^−1^.

Copper sorption was studied in batch experiments (pH 5.0) using different Cu concentrations ranging from 50 to 500 mg L^−1^ with different biosorbent concentrations of 0.5 to 2.5 g L^−1^ (Fig. 2). The highest removal rates of Cu (II) by dead biomass of *M. amorphae* CCNWGS0123 at different dosages, observed at an initial concentration of 100 mg L^−1^ Cu (II), were 37.0, 49.0, 53.0, 64.0 and 64.0%, respectively. Subsequently, the removal efficiency of copper decreased by increasing the initial copper concentration, while the lowest efficiency (9.0, 14.0, 16.0, 22.5 and 27.5%, respectively) was attained at the maximum copper concentration of 500 mg L^−1^. This may have been due to the high amount of metal ions that exceeded the available binding sites in *M. amorphae* from complexation of Cu (II) ions. This means that metal ions competing for the available binding site increased (45). Optimum concentration for the removal of copper by dead CCNWGS0123 was 100 mg L^−1^.

### Dosage

**Fig. 3:**
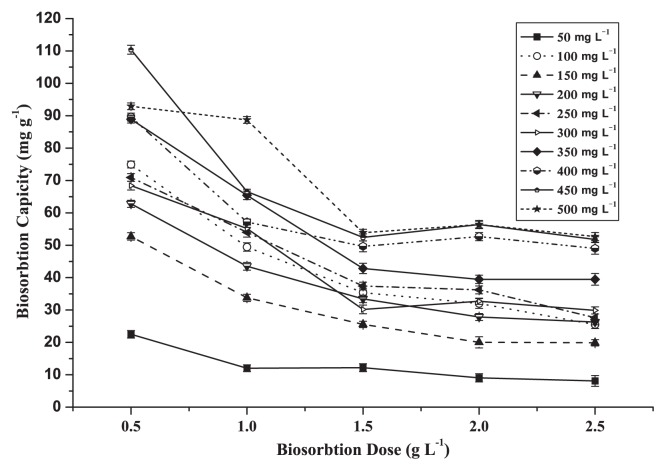
Biosorption capacity by dead cells of CCNWGS0123 at different dosage (0.5–2.5) g L^−1^ over initial copper concentration ranging from 50 to 500 mg L^−1^.

The amount of biosorbent dose used for this study is an important parameter as it determines the biosorption capacity (mg g^−1^), as shown in Fig. 3. The highest biosorption capacity was noted at 0.5 g L^−1^ at different initial copper concentrations of 22.5, 75.0, 53.0, 63.0, 71.0, 68.5, 89.0, 90.0, 110.0 and 93.0 mg g^−1^, respectively; however, the dosage of the biosorbent strongly influenced the extent of biosorption. In many instances, lower biosorbent dosage yielded higher uptake and lower removal efficiency (37). At the same time, the findings showed that by increasing the biomass concentration to 2.5 g L^−1^, the quantity of biosorbed solute per unit weight of biosorption decreased to 8.0, 25.5, 20.0, 26.0, 28.0, 30.0, 40.0, 49.0, 52.0 and 53.0 mg g^−1^ due to the complex interaction of several factors. The most important response was at the high sorbent dosage where the available solute was insufficient to completely cover the available exchangeable sites on the biosorbent, usually resulting in low solute uptake (34). Also, the electrostatic interaction increased with larger quantities of biomass (9). In addition, interference among binding sites due to increased biosorbent dosage can not be overruled, as this will result in low specific uptake (14). In this study, the optimum initial copper concentration and biosorption dose were 100 mg L^−1^ and 0.5 g L^−1^. Under these conditions, the following experiments were conducted.

### Effect of pH

**Fig. 4:**
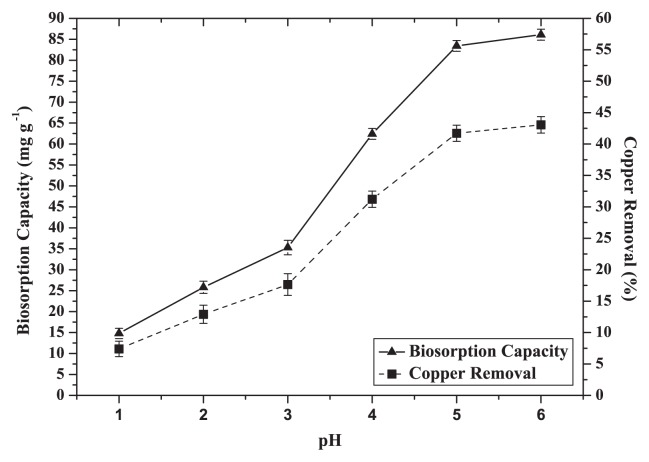
Effect of pH on biosorption capacity and removal efficiency of Cu^2+^ by dead cells of CCNWGS0123 (initial Cu^2+^ concentration: 100 mg L^−1^; temperature: 28°C; agitation speed: 150×*g*; biosorbent dose: 0.5 g L^−1^).

pH is an intrinsic factor that influences the biosorption process by affecting the solution chemistry of metals and the activity of functional groups of the biomass cell walls (15). It is noteworthy that the biosorption capacity and removal rate were low at the initial pH 1.0 but increased significantly from 13.0 mg g^−1^ (6.0%) to 73.0 mg g^−1^ (37.0%) until the pH reached 5.0. Metal uptake then remained almost stable at pH 6.0 at 74.0 mg g^−1^ (37.0%) (Fig. 4).

At low pH, the low biosorption uptake and removal efficiency could be attributed to the competition of the binding sites on CCNWGS0123 for hydrogen ions, so Cu cannot easily bond to the sites (44). Moreover, as pH increased to 5.0, more functional groups with negative charges, such as carboxyl, amine or hydroxyl, become exposed with the subsequent increase of attraction sites to positively charged ions, thus enhancing biosorption uptake. These results were similar to other research related to the biosorption of Cu (II) (4, 22). Based on this observation, the optimum pH for Cu absorption was set at 5.0 to avoid metal precipitation. Subsequent absorption experiments were performed at this pH value.

### Effects of agitation speed

**Fig. 5:**
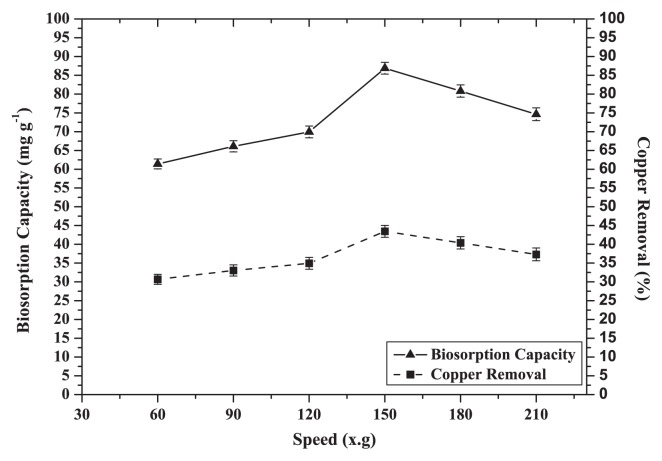
Effect of agitation speed on biosorption capacity and removal efficiency of Cu (II) by dead cells of CCNWGS0123 (initial Cu^2+^ concentration: 100 mg L^−1^; temperature: 28°C; pH 5.0; biosorbent dose: 0.5 g L^−1^).

Maximum biosorption capacity and removal efficiency increased from 61.4 mg g^−1^ (31.0%) to 87.0 mg g^−1^ (43.5%) respectively at 150×*g* (Fig. 5); however, increasing the agitation speed greater than 150×g decreased Cu (II) removal.

In some instances, external film diffusion can influence the rate of a biosorption process. Constant agitation can minimize this mass transfer resistance. Also, by increasing the agitation rate, the diffusion rate of a solute from the bulk liquid to the boundary layer liquid surrounding particles becomes higher due to enhanced turbulence as the thickness of the liquid boundary layer decreases (11). We found that biosorption of copper by CCNWGS0123 was more effective with moderate agitation (21, 35).

### Effects of temperature

**Fig. 6:**
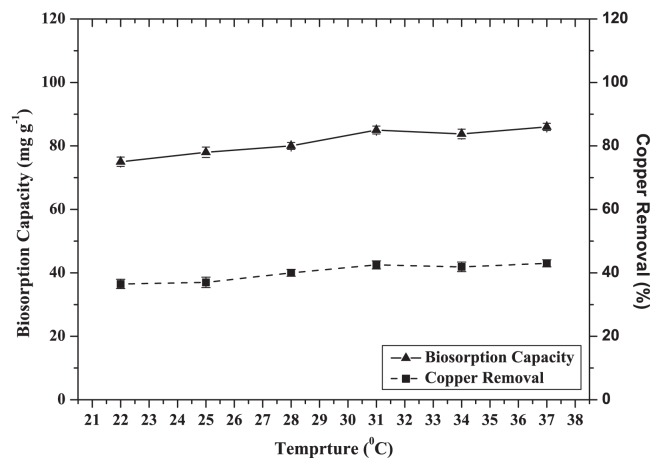
Effect of temperature on biosorption capacity and removal efficiency of Cu^2+^ by dead cells of CCNWGS0123 (initial Cu^2+^ concentration: 100 mg L^−1^; pH 5.0; agitation speed: 150×*g*; biosorbent dose: 0.5 g L^−1^).

Five temperatures were selected to study their influences on the biosorption of Cu (II) by dead CCNWGS0123. Results show that the maximum removal of Cu (II) was achieved between temperatures of 22 and 37°C (Fig. 6). There were no obvious differences in the biosorption capacity and removal rate at higher temperatures. This is in agreement with the results obtained by Yuan *et al.*(43). For the remaining experiments, the temperature of biosorption was 28°C because optimum growth of the strains occurred at 28°C. Previous experiments showed that the optimum conditions for Cu^2+^ biosorption by dead cells were 100 mg L^−1^ Cu (II), pH 5.0, agitated at 150×g and at dosage of 0.5 g L^−1^ biosorbent. The following biosorption experiments were conducted under these conditions.

### Biosorption isotherm

**Fig. 7:**
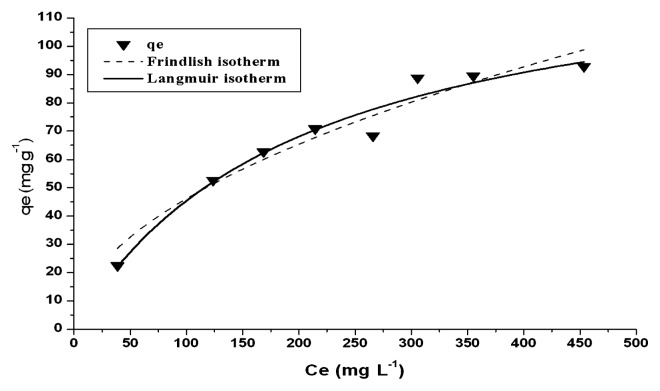
Langmuir and Freundlich isotherm fitting plots of biosorption of Cu (II) onto dead cells of CCNWGS0123, where qe is the equilibrium Cu^2+^ concentration on the biosorbent (mg g^−1^ dry cell) and Ce is the residual metal concentration in solution at equilibrium with the biosorbent (mg L^−1^).

Langmuir and Freundlich models have been used to describe biosorption isotherm. They are simple mathematical relationships characterized by a limited number of adjustable parameters, which give a good description of the experimental behavior over a large range of operating conditions (10). Nevertheless, these models do not reflect the mechanisms of sorbate uptake but they are capable of reflecting experimental curves (18) (Fig. 7).

The Langmuir model assumes a monolayer sorption process of a solute from a liquid solution (23). Furthermore, it is assumed that all the active sites on the sorbent surface have the same affinity by the sorbate (7). The Langmiur model can be represented as:

$${\text{q}}_{\text{e}}={\text{Q}}_{\text{max}}\;\text{b }{\text{C}}_{\text{e}}/1+\text{b }{\text{C}}_{\text{e}}$$

Where q_e_ is the equilibrium Cu^2+^ concentration on the biosorbent (mg g^−1^ dry cell); C_e_ is the residual metal concentration in solution at equilibrium with the biosorbent (mg L^−1^); Q_max_ indicates the maximum monolayer biosorption capacity of the biosorbent (mg g^−1^); and b is the Langmuir constant (L/mg) is related to the affinity of the binding sites.

The Freundlich isotherm was originally empirical in nature, and hypothesized as the heterogeneous energetic surface distribution of the active binding sites on the biomass. The general Freundlich equation is

$${\text{q}}_{\text{e}}={\text{K}}_{\text{f}}\;{{\text{C}}_{\text{e}}}^{1/\text{n}}$$

Where K_f_ is a constant related to biosorption capacity and 1/n is related to the effect of the concentration of metal ions (biosorption intensity) on the logarithmic plot of q_e_ versus C_e_, the slope of the plot is 1/n, and the intercept is K_f_. In this case, K_f_ can be used as a measure of the sorption capacity of the biosorbent for Cu^2+^, and represents the loading of Cu^2+^ on the solid surface when the equilibrium concentration of Cu^2+^ in solution is 1 mg L^−1^.

The experimental isotherm data of *M. amorphae* CCNWGS0123 resulting from the Langmuir and Freundlich plot are represented in Table 2, showing that the correlation coefficient of dead cells simulated by Langmuir was slightly higher (0.958) than that shown by Freundlich (0.936). This indicates that the data fit the Langmuir isotherm very well due to the homogenous distribution of active sites on the surface of CCNWGS0123 (1).

### Time course biosorption

**Fig. 8:**
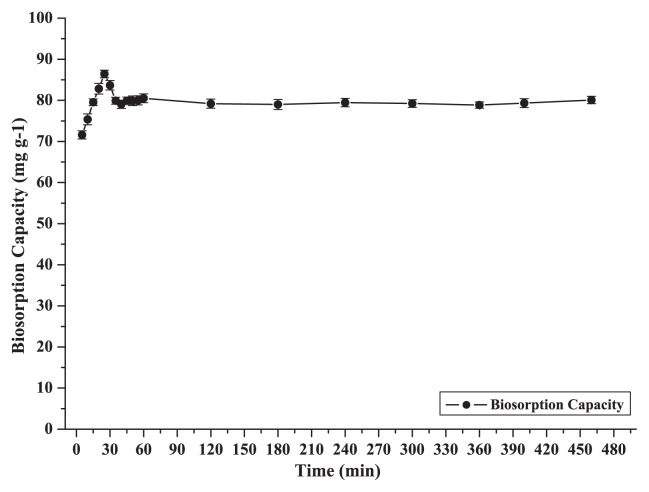
Effect of contact time on biosorption capacity of Cu^2+^ by dead cells of CCNWGS0123 (initial Cu^2+^ concentration: 100 mg L^−1^; temperature: 28°C; agitation speed: 150×*g*; biosorbent dose: 0.5 g L^−1^).

Effects of contact time at an initial copper concentration of 100 mg L^−1^ on the biosorption of Cu^2+^ by CCNWGS0123 are shown in Figure 8. The required time between the contact of absorbate and absorbent to reach equilibrium was 25 to 30 min, after which the biosorption capacity value showed no obvious changes for 24 h; however, this is quite normal as biosorption is considered to be a spontaneous process and similar results have been reported (43, 45).

### FT-IR analysis

**Fig. 9:**
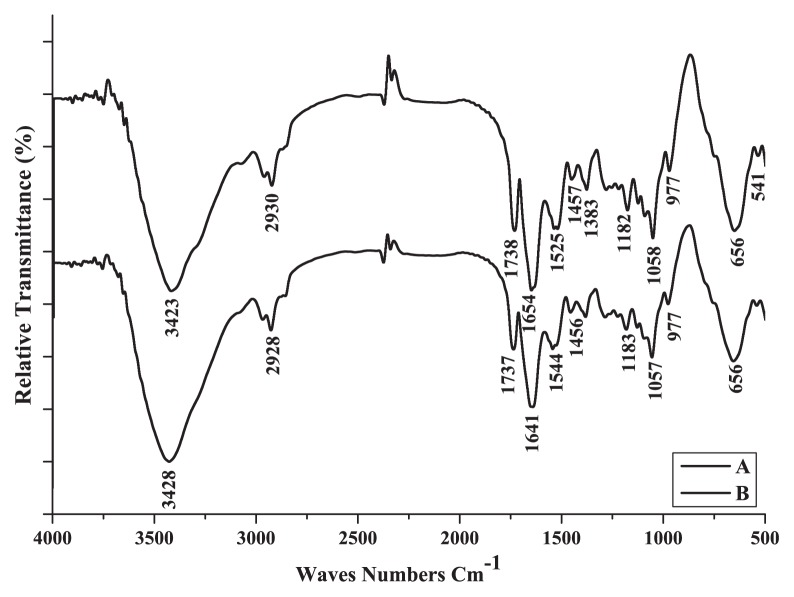
FT-IR spectrum of dead biosorbent loaded with and without Cu (II). (A) natural dead CCNWGS0123. (B) Cu^2+^-loaded dead CCNWGS0123 100 mg L^−1^.

FT-IR analysis was carried out on the dried biosorbent cells in order to determine the functional groups involved in the metal binding before and after the biosorption process of Cu^2+^ by dead CCNWGS0123 (Fig. 9A, B). The IR spectrum of dead biomass showed distinct and strong bonds by the number of peaks reflecting the complex nature of the *M. amorphae* cell surface. Hence, there was a change in the intensity of the bands in different regions after interaction with Cu^2+^. The stretching vibration of hydrogen bonds in hydroxyl -OH or amine -NH groups shifted from 3,423 to 3,428 cm^−1^. The phenolic or carboxylic groups shifted from 2,930 to 2,928 cm^−1^. The peaks shifted from 1,738 to 1,737 cm^−1^, mainly C=O stretch. In particular, amide I groups shifted from 1,654 to 1,641 cm^−1^. In addition, strong biosorption peaks of amide II groups shifted from 1,525 to 1,544 cm^−1^. It was clear that carboxylate ions shifted from 1,457 to 1,456 cm^−1^. A band at about 1,182 cm^−1^ related to amide III stretching shifted to 1,183 cm^−1^. The peak representing the C-N, C-O groups shifted from 1,058 to 1,057. Furthermore, particular absorption bonds for aromaticalkyl and alkene structures were obtained below 1,000 cm^−1^(977, 656, 465) cm^−1^, as reported previously (31).

The FT-IR spectrum transmittance of Cu^2+^ before and after biosorption by CCNWGS0123 showed complex and additive impacts of chemical structures composed of different functional groups, such as O-H, N-H, C-H, C=O, -NH, -CN, C-N, C-O, amide I, II, III and unsaturated alkenes, alkyls and aromatic groups on the cell surface, indicating the possibility of cell-cation interactions such as H-bonding, complexation, etc., as reported previously (2, 21).

### SEM/EDX analysis

**Fig. 10:**
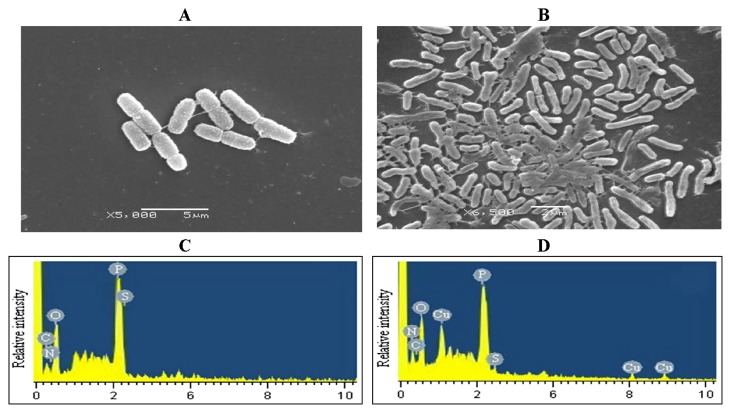
SEM micrographs and EDX spectra of CCNWGS0123. A: Control for SEM; B: 100 mg L^−1^ Cu^2+^ loaded for SEM; C: Control for EDX spectra; D: 100 mg L^−1^ Cu^2+^-loaded for EDX spectra.

Surface characterization of the cells loaded with 100 mg L^−1^ Cu (II) was performed. Scanning electron microscopy is an extremely useful tool for visual confirmation of surface morphology and the physical state of the surface (Fig. 10A, B). We observed irregular and aggregated cellular morphology in Cu^2+^-loaded cells. Deformation and cell-surface damage may be due to the secretion of extracellular polymeric substances during metal biosorption (5). Moreover, EDX analysis (Fig. 10C, D) of Cu^2+^-loaded biomass revealed three distinct peaks at 1.1 keV, 8.1 keV and 8.9 keV, implying that copper ions have been absorbed on the cells (30).

## Conclusions

In conclusion, our studies showed that dead cells of *Mesorhizobium amorphae* CCNWGS0123 are an effective absorbent for copper removal from aqueous solution. The removal of Cu^2+^ increased significantly by increasing the pH up to 5.0, agitation speed 150×*g* and temperature 28°C. Equilibrium was reached within 30 min. The absorption isotherms could be well fitted by the Langmuir equation followed by the Freundlich equation. The highest removal of initial copper concentration was achieved at a dosage of 100 mg L^−1^ and the highest biosorption capacity was found at an initial concentration of 0.5 g L^−1^ Cu (II). The IR spectral data indicated that many function groups were involved in the removal process of dead absorbents and the sorption data were supported by SEM/EDX. To our knowledge, this is the first time that dead cells of CCNWGS0123 have been used as an absorbent of copper ions. Based on our results, we foresee the possibility of using dead cells of *Mesorhizobium amorphae* CCNWGS0123 for the removal of Cu ions from aqueous solution compared with other biomaterials, thus reducing aqueous environmental pollution.

## Figures and Tables

**Fig. 1:**
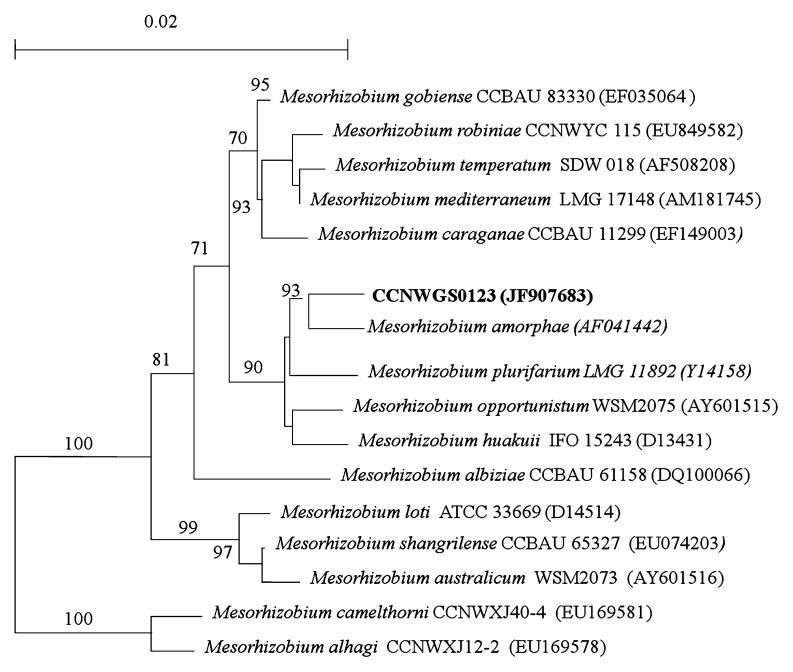
Phylogenetic tree based on 16S rRNA gene of CCNWGS0123 and reference strains with neighbor-joining method (numbers at nodes indicate bootstrap values. 0.02 denotes the genetic distance).

**Table 1:** Oligonucleotides used as PCR and sequencing primers

Target genes	Primer Sequence	Amplification Size	PCR annealing temperature	References
16S rRNA	CGG GAT CCA GAG TTT GAT CCT GGC TCA GAA CGA ACG CT CGG GAT CCT ACG GCT ACC TTG TTA CGA CTT CAC CCC	1379 bp	56°C	(17)
*nodA*	TGC RGT GGA RDC TRY GCT GGG AAA GGN CCG TCR TCR AAS GTC ARG TA	622 bp	57°C	(19)
*nifH*	GGN ATC GGC AAG TCS ACS AC TCR AMC AGC ATG TCC TCS AGC TC	774 bp	58°C	(35)
*NodC*	AYG THG TYG AYG ACG GTT C CGY GAC AGC CAN TCK CTA TTG	900 bp	53°C	(42)

**Table 2:** Langmuir and Freundlich isotherm constants of Cu (II) on dead CCNWGS0123

Strain	Langmuir constants			Freundlich constants		

	Q_max_ (mg g^−1^)	b (mg L^−1^)	R^2^	K_f_ ([mg g^−1^][mg L^−1^]^n)^	1/n	R^2^
Dead	136.02	0.0050	0.958	4.546	0.503	0.936
